# Discriminating atypical parotid carcinoma and pleomorphic adenoma utilizing extracellular volume fraction and arterial enhancement fraction derived from contrast‐enhanced CT imaging: A multicenter study

**DOI:** 10.1002/cam4.7407

**Published:** 2024-06-20

**Authors:** Zhen‐Yu Xu, Lin‐Wen Huang, Yun‐Jun Yang, Zhi‐Ping Cai, Mei‐Lin Chen, Rui‐Liang Lu, Yong‐Xi Ouyang, Zhen‐Kai Hong, Wei‐Jun Huang, Zhi‐Feng Xu

**Affiliations:** ^1^ Department of Radiology The First People's Hospital of Foshan Foshan China; ^2^ Department of Radiology Shunde Hospital, Southern Medical University (The First People's Hospital of Shunde) Foshan China; ^3^ Department of Ultrasound The First People's Hospital of Foshan Foshan China

**Keywords:** arterial enhancement fraction, computed tomography, extracellular volume fraction, parotid carcinoma, pleomorphic adenoma

## Abstract

**Objectives:**

To investigate the added value of extracellular volume fraction (ECV) and arterial enhancement fraction (AEF) derived from enhanced CT to conventional image and clinical features for differentiating between pleomorphic adenoma (PA) and atypical parotid adenocarcinoma (PCA) pre‐operation.

**Methods:**

From January 2010 to October 2023, a total of 187 cases of parotid tumors were recruited, and divided into training cohort (102 PAs and 51 PCAs) and testing cohort (24 PAs and 10 atypical PCAs). Clinical and CT image features of tumor were assessed. Both enhanced CT‐derived ECV and AEF were calculated. Univariate analysis identified variables with statistically significant differences between the two subgroups in the training cohort. Multivariate logistic regression analysis with the forward variable selection method was used to build four models (clinical model, clinical model+ECV, clinical model+AEF, and combined model). Diagnostic performances were evaluated using receiver operating characteristic (ROC) curve analyses. Delong's test compared model differences, and calibration curve and decision curve analysis (DCA) assessed calibration and clinical application.

**Results:**

Age and boundary were chosen to build clinical model, and to construct its ROC curve. Amalgamating the clinical model, ECV, and AEF to establish a combined model demonstrated superior diagnostic effectiveness compared to the clinical model in both the training and test cohorts (AUC = 0.888, 0.867). There was a significant statistical difference between the combined model and the clinical model in the training cohort (*p* = 0.0145).

**Conclusions:**

ECV and AEF are helpful in differentiating PA and atypical PCA, and integrating clinical and CT image features can further improve the diagnostic performance.

## 1 INTRODUCTION

Salivary gland tumors represent a relatively uncommon occurrence, constituting approximately 3%–6% of head and neck tumors, with 80% of these tumors manifesting in the parotid gland, wherein about 20% exhibit malignant characteristics.^1^, ^2^ Surgical resection remains the primary treatment modality for parotid tumors (PTs); however, diverse pathological types necessitate distinct treatment strategies, leading to considerable variations in prognosis. Among benign tumors, PA exhibits the highest incidence.^3^ Clinical interventions, such as partial or total parotidectomy, are commonly employed, while PCA mandates adjuvant radiotherapy and chemotherapy post radical resection.^4^, ^5^ Consequently, achieving precise preoperative diagnosis is imperative. Although fine‐needle aspiration cytology (FNAC) is a pivotal method for diagnosing PTs preoperatively, its accuracy can be compromised by the tumor's location and inadequate sampling. Despite the enhanced diagnostic accuracy provided by ultrasound‐guided core biopsy (USCB), concerns persist regarding the potential for facial nerve injury and tumor cell dissemination along the needle tract. Additionally, ultrasound examination of deep‐seated tumors faces significant limitations.^6^ In a study by Suzuki et al.^7^ involving 996 patients, FNAC demonstrated a correct rate of 72.0% for benign PTs; however, when considering both benign and malignant tumors, the diagnostic accuracy dropped to 55.5%. CT and MR imaging techniques have become integral in diagnosing, grading, and prognosticating PTs, with MR being the preferred choice due to its superior soft tissue resolution. Various functional MRI techniques, such as FE‐T2WI, DWI, dynamic contrast‐enhanced T1‐weighted imaging(DCE‐T1WI), Arterial spin labeling(ASL), and Intra‐voxel incoherent motion(IVIM), have been explored for evaluating PTs.^8^, ^9^, ^10^, ^11^ Nevertheless, the interpretation of imaging features is constrained by the expertise of diagnosticians and is inherently subjective. Furthermore, differences in scanning protocols pose challenges to result stability and repeatability. The optimal threshold for distinct quantitative indicators in discriminating PT types remains undetermined, contributing to ongoing controversies regarding its clinical utility. Despite concerns of radiation injury, CT is widely employed in clinical practice for evaluating PTs due to its cost‐effectiveness. Notably, PA and PCA, particularly in the early stages, often present with overlapping clinical and imaging manifestations, such as a predilection for middle‐aged and elderly women, painless parotid mass, and delayed enhancement in enhanced CT imaging.^12^, ^13^ Consequently, traditional CT techniques encounter challenges in achieving precise diagnoses for both entities. While recent years have witnessed considerable advancements in radiomics for PT evaluation,^14^ manual labeling, specialized analysis software, and high‐speed processors contribute to elevated human and financial costs. Moreover, due to the rarity of PTs and the absence of robust data support, the comprehensive diagnostic efficiency, stability, and robustness of these models remain insufficient, yielding controversial outcomes in clinical application.

Extracellular volume (ECV) encompasses the summation of extravascular‐extracellular space and the fraction occupied by intravascular space, offering insights into microvessel density (MVD) and the extent of matrix fibrosis. It serves as a quantifiable measure of the extracellular matrix (ECM), thereby providing a reflection of the tumor microenvironment.^15^ In contrast to traditional biopsy methods, ECV offers a non‐invasive, convenient, and highly reproducible approach. Previous investigations have underscored the comparable diagnostic efficacy of ECV and biopsy.^16^ In clinical contexts, the utility of ECV scores has been validated in assessing the pathological characteristics of cardiac and liver fibrosis.^17^, ^18^, ^19^ Studies by Takumi et al.^20^ revealed an inverse relationship between the degree of differentiation of thymic epithelial tumors on CT and ECV values. Higher ECV values corresponded to lower degrees of differentiation, enabling a more accurate diagnosis of pathological tissue types in thymic malignant tumors.^21^ Additionally, Adams et al.^22^ demonstrated the potential of ECV values obtained through magnetic resonance imaging in grading clear cell renal cell carcinoma pathologically. Arterial enhancement fraction (AEF) denotes the ratio of the absolute enhancement increment during the arterial phase to that in the venous phase. Prior investigations have established the utility of AEF in detecting liver fibrosis, diagnosing hepatocellular carcinoma, and predicting the probability of recurrence in liver cancer and liver metastases.^23^, ^24^, ^25^, ^26^ Notably, within the current research landscape, there is a dearth of reports on the application of ECV and AEF in evaluating parotid gland tumors. Our prior studies have delineated distinct pathological characteristics between PA and PCA. PA exhibits a higher content of mucinous stroma and sparse capillary networks, while PCA cells are densely arranged with abundant capillary networks. Notably, their arterial phase enhancement tends to surpass that of PA.^27^ Consequently, we posit that ECV and AEF hold promise in enhancing the precision of differential diagnoses between PCA and PA.

The primary objective of this investigation is to assess the utility of ECV and AEF in the preoperative differentiation between PA and atypical PCA. Additionally, to integrate clinical and CT features to formulate a combined model, thereby enhancing the overall diagnostic efficacy.

## 2 MATERIALS AND METHODS

### 2.1 Patients

Institutional ethics review board approval was obtained and the requirement for informed consent was waived for this retrospective study.

**FIGURE 1:**
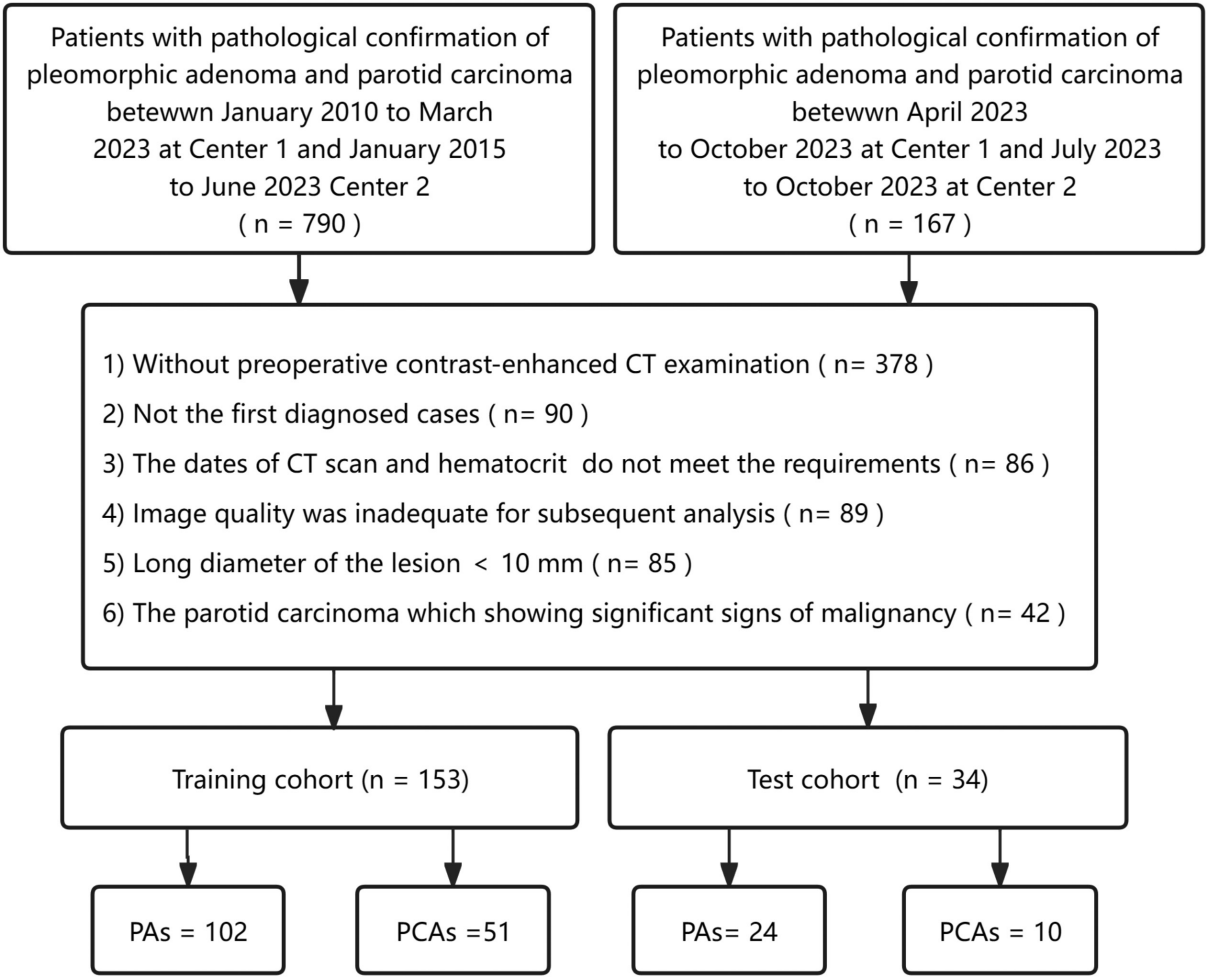
Flowchart of patient enrolment.

This study compiled clinical and CT imaging data from surgically and pathologically confirmed cases of PA and PCA, covering the period from January 2010 to October 2023 in the first center and January 2015 to October 2023 in the second center. Inclusion criteria comprised: (1) Initial diagnosis of parotid gland tumor; (2) availability of complete preoperative CT dynamic enhanced scan images; (3) time interval of within 2 weeks between CT examination and surgery; (4) Time interval of within 1 week between routine blood test and CT examination. Exclusion criteria included: (1) Poor image quality that impedes subsequent analysis; (2) lesion long diameter measuring less than 10 mm; (3) history of prior head and neck tumors and corresponding treatments; (4) clear indications of malignancy in parotid gland cancer, such as invasion of adjacent tissues or lymph nodes indicating metastasis. In total, 187 patients met the criteria for inclusion in this study, comprising 126 cases of atypical PAs and 61 cases of PCAs. The flowchart of patient enrolment is shown in Figure 1.

### 2.2 CT examination

Parotid gland scans were conducted using Philips Brilliance iCT 256‐slice spiral CT, GE Revolution 256‐slice CT, and GE Discovery 64‐slice CT machines, encompassing both plain scans and multi‐phase enhanced scans. The scanning parameters were set at 120 kV, 250 mA, and a layer thickness of 3–5 mm. The scanning range extended from the infraorbital edge to the level of the upper clavicle edge, ensuring comprehensive coverage of the entire parotid gland. Following the acquisition of plain scan images, the contrast agent iohexol (300 mgL/mL) was intravenously injected through the cubital vein at a flow rate of 3.0–3.5 mL/s. Subsequently, arterial and venous phases were obtained at 35–40 s and 65–70 s post‐contrast agent injection, respectively. Patients were instructed to refrain from swallowing and making head movements during the examination.

### 2.3 CT image analysis

All CT images underwent independent assessment by two radiologists, possessing 3 and 8 years of experience in cervical radiological diagnosis, respectively. Both radiologists were blinded to the patients' pathological diagnoses during the evaluation process. Various CT features of the tumors were meticulously evaluated, including location (with deep lobe/without deep lobe), length (mm), calcification (present/absent), boundary (clear/unclear), shape (regular/irregular), necrosis or cystic changes (present/absent), and Vaso‐welt sign (present/absent). Assessment of all morphological characteristics was conducted on venous phase images, allowing for the qualitative adjustment of window level or width. Intralesional necrosis or cystic changes were defined as areas exhibiting attenuation values<30 HU during the venous phase. Any disparities in the radiologists' evaluations were resolved through consensus. For quantitative assessment, the largest layer of the lesion was selected to delineate the region of interest (ROI), and the CT value (HU) was measured. This process ensured the exclusion of any internal necrotic or hemorrhagic areas during outlining. Subsequently, the CT value of the lesion was measured at each scanning stage. Additionally, the CT value of the blood pool was determined by obtaining the ROI in the ipsilateral external carotid artery area, avoiding the vessel wall, which included any wall calcification and thrombus attached to the wall. The largest area of the ROI was measured, and the average value, derived from three measurements, was considered the final value. Representative values for all quantitative variables for each tumor were obtained as the average of the two radiologists' assessments.

The Extracellular volume (ECV) fraction (%) was determined utilizing the following formula:

$$\mathrm{ECV}\;\left(\%\right)=\left(1-\text{hematocrit}\right)\times \left(\text{CELesion}/\text{CEAorta}\right)\times 100\%,$$

where CELesion denotes the disparity between the average CT value during the venous phase of the focal lesion and the average CT value during the plain scan phase (in Hounsfield Units, HU), and CEAorta represents the difference between the venous phase (HU) of the ipsilateral external carotid artery and the plain scan phase (HU).

The arterial enhancement fraction (AEF) (%) is computed using the following formula:

$$\mathrm{AEF}\;\left(\%\right)=\left(\mathrm{HUa}–\mathrm{HUu}\right)/\left(\mathrm{HUv}–\mathrm{HUu}\right)\times 100\%,$$

wherein HUu, HUa, and HUv signify the average CT values (in HU) during the plain scan phase, arterial phase, and venous phase of the tumor at the corresponding level.

### 2.4 Model construction and evaluation of diagnostic performance

The finalized cases underwent single‐factor analysis to identify parameters exhibiting statistical differences. Subsequently, binary logistic regression was applied to identify independent risk factors. Clinical parameters and CT characteristics identified as independent predictors were amalgamated to formulate a clinical model. ECV, AEF, and clinical models were then integrated to create a combined model. The diagnostic performance of the models constructed by ECV, AEF, the clinical model, ECV and AEF in combination with the clinical model, and the combined model were evaluated in both the training cohort and test cohort using Receiver Operating Characteristic (ROC) curves. Calibration curve and Decision Curve Analysis (DCA) were employed to assess the calibration and clinical applicability of the combined model. The Hosmer–Lemeshow test was utilized to scrutinize the fit between the predicted values of the combined model and the observed values.

### 2.5 Statistical analysis

**FIGURE 2:**
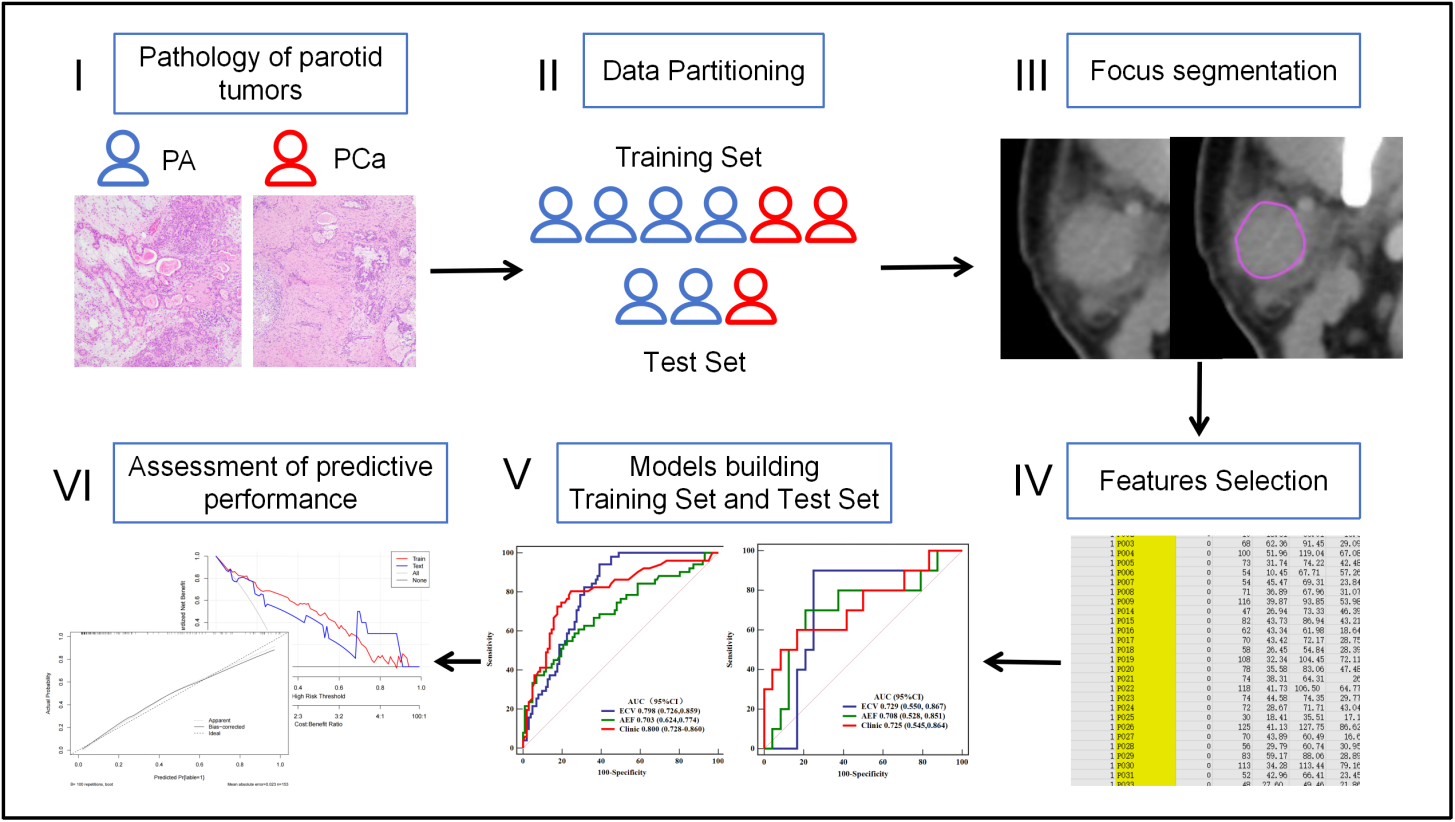
Schematic diagram of the full proposed method.

Statistical analyses were conducted using SPSS version 26.0, MedCalc software, and R statistical software (version 3.6.3). Descriptive statistics for measurement data were presented as mean ± standard deviation, and the independent samples *t*‐test or Wilcoxon rank‐sum test was employed as appropriate. Categorical variables were analyzed using the chi‐squared test or Fisher's exact probability method. Logistic regression analysis was performed on clinical characteristic indicators exhibiting statistically significant differences, and parameters with such differences were incorporated into model construction. In both the training and test cohort, the diagnostic performance of each model was assessed through ROC curve analysis, and the area under the curve (AUC) was calculated. The Nomogram plot of the combined model was generated using R statistical software. Model calibration was evaluated through fit tests on the ROC curve, and the performance of the clinical prediction model was further assessed using DCA. The schematic diagram of the full proposed method is shown in Figure 2.

## 3 RESULTS

### 3.1 Baseline data of patients

This study comprised a total of 187 patients, with 153 individuals constituting the training cohort (102 PAs and 51 atypical PCAs) and 34 individuals forming the test cohort (24 PAs and 10 atypical PCAs). Univariate analysis conducted in the training cohort revealed a statistically significant difference in age (*p* = 0.024), whereas the difference in gender did not reach statistical significance (*p* = 0.818) (Table 1).

**TABLE 1:** Comparison of clinical and CT imaging features.

	Training cohort (*n* = 153)		*p* value	Test cohort (*n* = 34)		*p* value
	PA (*n* = 102)	PCa (*n* = 51)		PA (*n* = 24)	PCa (*n* = 10)	
Gender						
Male	44 (43.14%)	23 (45.09%)	0.818	11 (45.83%)	7 (70.00%)	0.198
Female	58 (56.86%)	28 (54.90%)		13 (54.17%)	3 (30.00%)	
Age	42.58 ± 13.07	48.92 ± 17.33	0.024*	52.2 ± 10.26	45.33 ± 8.66	0.255
HCT	0.413 ± 0.046	0.420 ± 0.050	0.4	0.416 ± 0.041	0.436 ± 0.024	0.163
ECV%	21.75 ± 11.52	34.10 ± 10.74	<0.001*	22.00 ± 13.99	30.11 ± 8.13	0.097
AEF%	48.16 ± 25.12	72.46 ± 36.49	<0.001*	44.87 ± 16.67	56.75 ± 18.18	0.074
Long diameter (mm)	24.15 ± 8.44	28.16 ± 10.17	0.011*	26.33 ± 8.45	26.40 ± 9.14	0.984
Irregular shape	65 (63.72%)	40 (78.43%)	0.065	12 (50.00%)	3 (30.00%)	0.285
Location						
With deep lobe	41 (40.20%)	29 (56.86%)	0.117	8 (33.33%)	7 (70.00%)	0.139
Without deep lobe	61 (59.80%)	22 (43.14%)		16 (66.67%)	3 (30.00%)	
Calcification	3 (2.94%)	7 (13.72%)	0.011*	0 (0.00%)	1 (90.00%)	0.116
Boundary	86 (84.31%)	18 (35.29%)	<0.001*	18 (75.00%)	6 (60.00%)	0.382
Necrotic or cystic	58 (56.86%)	30 (58.82%)	0.817	16 (66.67%)	4 (40.00%)	0.150
Vaso‐welt sign	9 (8.82%)	13 (25.49%)	0.006*	1 (41.67%)	1 (10.00%)	0.510

Abbreviations: AEF, arterial enhancement fraction; ECV, extracellular volume; PA, pleomorphic adenoma; PCa, parotid carcinoma.

* *p* < 0.05.

### 3.2 Clinical and CT imaging features of PAs and PCAs

**FIGURE 3:**
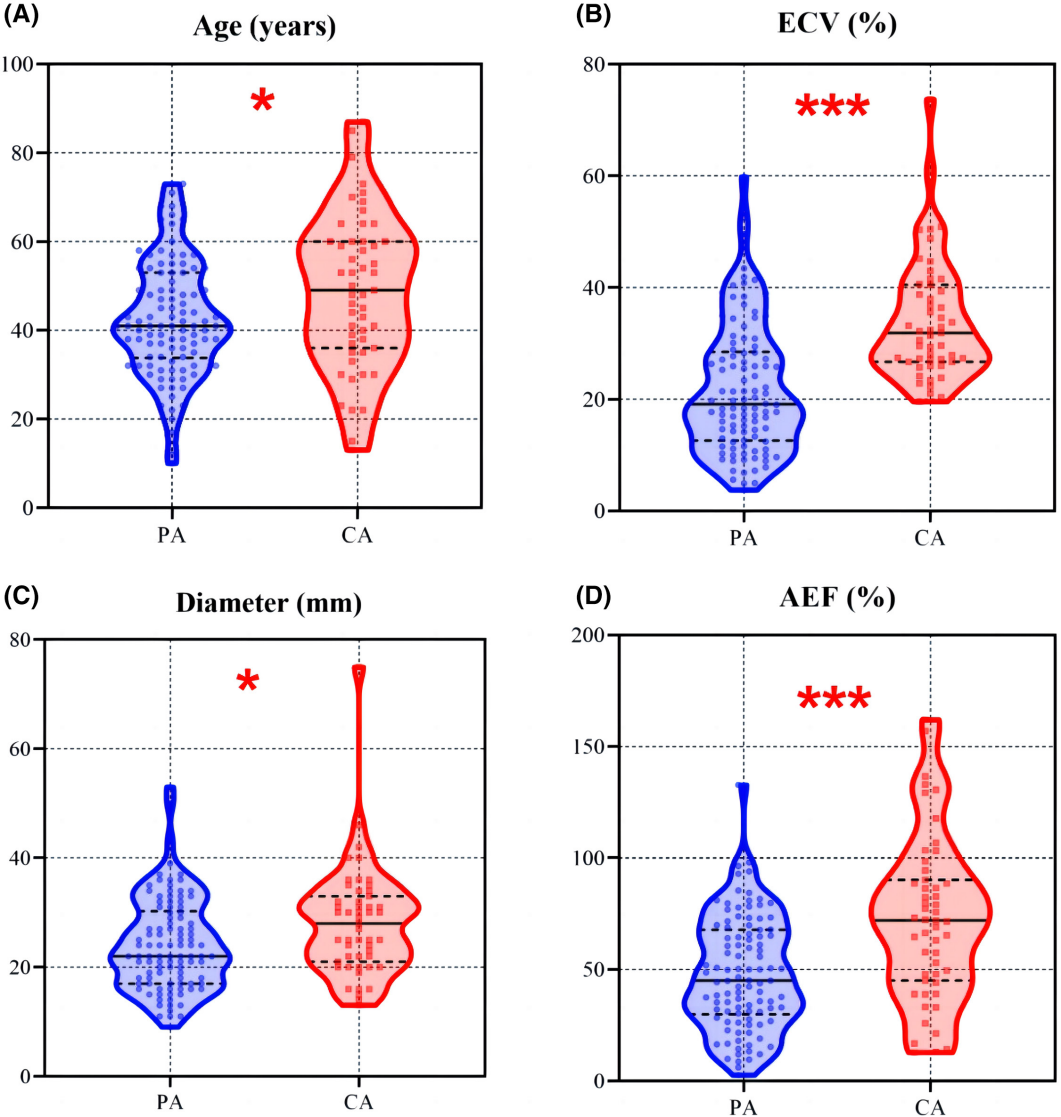
Violin plot of Age (A), ECV (B), Diameter (C), and AEF (D) in the training cohort. ***p < 0.001; *p < 0.05.

Table 1 provides a comprehensive summary of the results obtained from the univariate analysis of clinical characteristics and CT features. Within the training cohort, statistically significant differences were observed between PA and atypical PCA in parameters such as ECV, AEF, long diameter, calcification, boundary, and vascular welting. The distribution of ECV, AEF, age, and long diameter within the training cohort is visually depicted in the violin plot presented in Figure 3. Conversely, no statistically significant differences (*p* > 0.05) were detected in other clinical and CT features. For the training cohort, binary logistic regression analysis incorporated variables including ECV, AEF, age, length, calcification, boundary, and vessel welting (Table 2). The results indicated that ECV (*p* = 0.002, 95% CI: 1.271–465.333, OR = 1.07, diagnostic threshold: 22.14), AEF (*p* < 0.001, 95% CI: 0.899–6.187, OR = 1.035, diagnostic threshold: 65.18), age (*p* = 0.022, 95% CI: 1.292–51.205, OR = 1.038, diagnostic threshold: 57), and boundary (*p* < 0.001, 95% CI: 0.998–1.006, OR = 10.166) emerged as inde*p*endent predictors for distinguishing between PA and atypical PCA.

**TABLE 2:** Results of multivariate logistic regression analysis.

Characteristic	*β*	SE	Wald *χ* ^2^	OR	95% CI	*p* value
ECV	0.068	0.022	9.21	1.07	1.271–465.333	0.002*
AEF	0.035	0.009	14.169	1.035	0.899–6.187	<0.001*
Age	0.037	0.016	5.242	1.038	1.292–51.205	0.022*
Long diameter (mm)	0.008	0.032	0.068	1.008	0.480–22.712	0.794
Calcification	−1.616	1.048	2.376	0.199	0.899–6.187	0.123
Boundary	2.319	0.525	19.482	10.166	0.998–1.006	<0.001*
Vaso‐welt sign	−0.336	0.689	0.239	0.714	0.999–1.015	0.625

Abbreviations: AEF, arterial enhancement fraction; ECV, extracellular volume.

* *p* < 0.05.

### 3.3 Interobserver agreement

The inter‐observer agreement for qualitative assessment was excellent (0.983 for calcification, 0.979 for margin, and 0.928 for vessel welt); the inter‐observer agreement for quantitative parameter assessment was good (0.977 for long diameter, 0.813 for ECV score, and 0.834 for AEF).

### 3.4 Model construction and assessment of predictive performance

**FIGURE 4:**
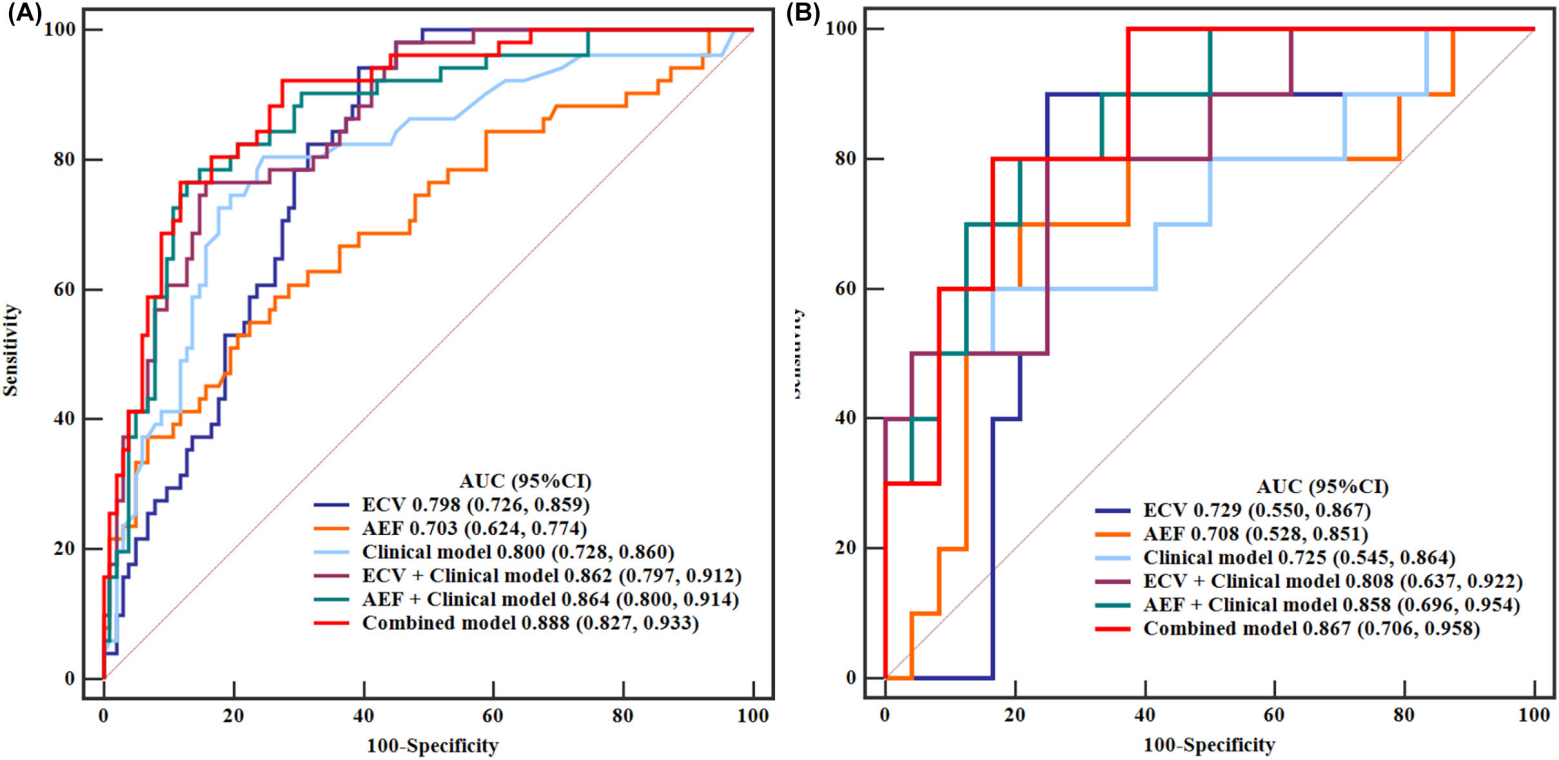
The ROC curves of each model are summarized. Figure 4A represents the ROC curve of each model in the training cohort; Figure 4B represents the ROC curve of each model in the test cohort.

Develop a clinical model based on age and boundary, and construct its ROC curve. Illustrate the ROC curves for ECV and AEF individually, and in combination with the clinical model. Subsequently, amalgamate the clinical model, ECV, and AEF to establish a comprehensive combined model and delineate its ROC curve. Notably, the combined model exhibited superior diagnostic performance in both the training cohort and the test cohort (training cohort AUC = 0.888, 95% CI: 0.827–0.933; test cohort AUC = 0.867, 95% CI: 0.706–0.958). Detailed differential diagnostic performance metrics for each model are presented in Table 3. The ROC curves for various models in both the training cohort and test cohort are depicted in Figure 4. Delong's test revealed a significant statistical difference between the combined model and the clinical model in the training cohort (training cohort: *Z* = 2.444, *p* = 0.0145; test cohort: *Z* = 1.520, *p* = 0.1285), underscoring the substantial contributions of integrating ECV and AEF to the construction of the combined model.

**TABLE 3:** Differential diagnostic efficacy of models in training cohort and test cohort.

Model	Training cohort (*n* = 153)				Text cohort (*n* = 34)			
	AUC (95% CI)	Sensitivity (%)	Specificity(%)	*p* value	AUC (95% CI)	Sensitivity (%)	Specificity (%)	*p* value
Clinical model	0.800 (0.728, 0.860)	80.39	75.49		0.725 (0.545, 0.864)	60	83.33	
ECV	0.798 (0.726, 0.859)	94.12	60.78	0.9734	0.729 (0.550, 0.867)	90	75%	0.9771
AEF	0.703 (0.624, 0.774)	58.82	73.53	0.1552	0.708 (0.528, 0.851)	70	79.17	0.9273
ECV + clinical model	0.862 (0.797, 0.912)	76.47	84.31	0.0232*	0.808 (0.637, 0.922)	80	75.00	0.0832
AEF + clinical model	0.864 (0.800, 0.914)	78.43	85.29	0.0704	0.858 (0.696, 0.954)	80	79.17	0.1856
Combined model	0.888 (0.827, 0.933)	92.16	72.55	0.0145*	0.867 (0.706, 0.958)	80	83.33	0.1285

Abbreviations: AEF, arterial enhancement fraction; ECV, extracellular volume.

* *p* < 0.05.

**FIGURE 5:**
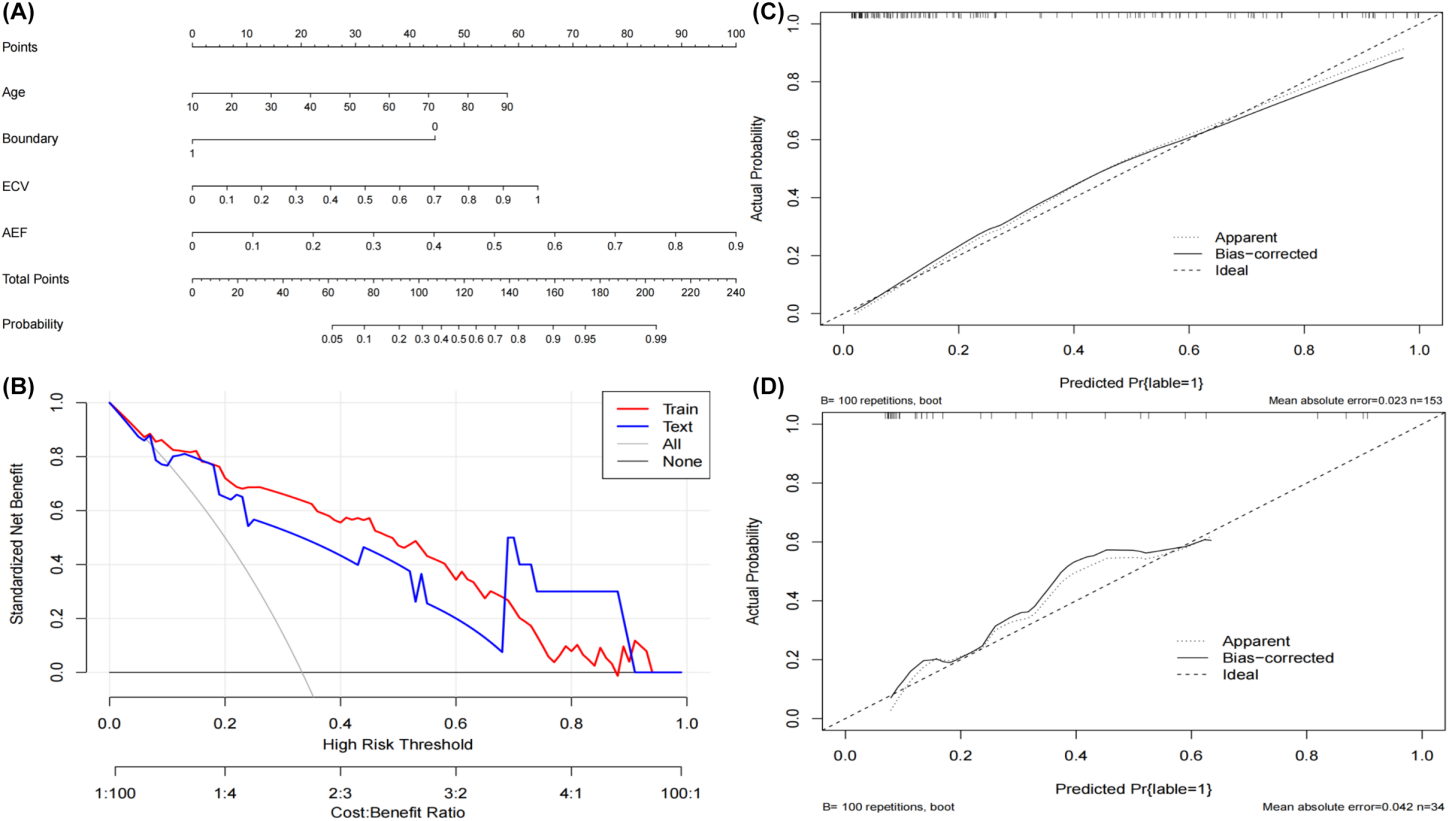
Display of nomogram (5A), DCA curves (5B) and calibration curves of training cohort (5C) and test cohort (5D).

**FIGURE 6:**
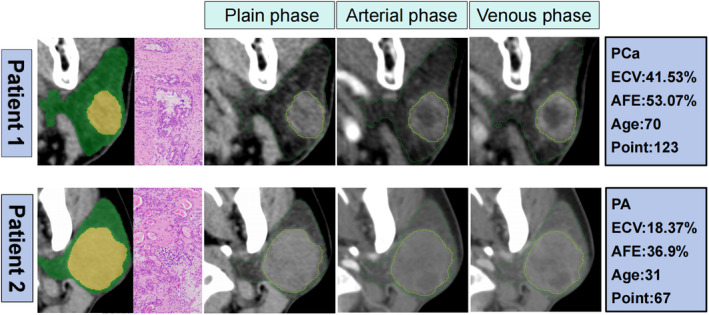
The representative cases of PA and atypical PCA.

The Nomogram, constructed based on independent risk factors, is presented in Figure 5A,B which display the DCA decision curves for the training cohort and the test cohort, demonstrating the commendable diagnostic efficiency of the combined model in both cohorts. Calibration curves for the combined model are depicted in Figure 5C,D. The calibration curve results reveal a strong concordance between predicted observed values and actual observed values in the training cohort. Furthermore, the Hosmer–Lemeshow test indicates that the differences between the training cohort and the test cohort of the combined model are not statistically significant (*p* = 0.644, 0.214), underscoring the robust fit of the established combined model with real data. Figure 6 showcases representative cases of PA and atypical PCA.

## 4 DISCUSSION

This study assesses the preoperative identification efficacy of ECV and AEF for PA and atypical PCA by developing clinical feature models, ECV models, and AEF models, as well as a combined model. The findings reveal that the combined model exhibits the highest diagnostic performance in both the training cohort and the test cohort (AUC = 0.888 and 0.867). Notably, the difference between the training cohort and the clinical feature model is statistically significant. This underscores the substantial improvement in diagnostic performance achieved by integrating ECV and AEF into the model.

The survival and growth of solid tumor cells are significantly influenced by factors such as the extracellular matrix and blood vessel growth, which play pivotal roles in the development of malignant tumors and are closely associated with tumor invasiveness.^28^ The extracellular matrix (ECM), as an integral component of the cellular microenvironment, undergoes changes during tumor occurrence and progression. ECV as a quantitative measure of the ECM, reflects alterations in the cellular microenvironment. Numerous studies have validated the consistency between ECV obtained through CT imaging and the ECM observed in biopsy specimens, affirming the viability of calculating CT values and applying ECV.^29^, ^30^ Microvessel density (MVD), on the other hand, is intricately linked to tumor differentiation. The accelerated growth of tumor blood vessels correlates with increased malignancy, heightened blood supply, adverse patient prognosis, and a correspondingly elevated MVD.^31^, ^32^ In this study, ECV derived from CT image measurements emerged as an independent predictor for distinguishing PA and atypical PCA. It exhibited exceptional diagnostic value in both the training and test cohorts, with AUCs of 0.798 and 0.72, respectively. The research findings indicate that PCA manifests higher ECV than PA, indicative of lower differentiation and a poor prognosis. This observation aligns with Takumi et al.'s discovery^20^ that thymic epithelial tumors exhibit lower ECV on CT images. The parallel conclusion is consistent with Li et al.'s investigation,^33^ where statistically significant differences in ECV, based on CT images, were noted between high‐grade and low‐grade rectal adenocarcinoma. Higher ECV values correlated with lower degrees of differentiation and higher pathological grades. These studies collectively underscore the prognostic value of ECV in predicting tumor differentiation.

Furthermore, AEF denotes the ratio of the absolute enhancement in the arterial phase to that in the venous phase, serving as an indirect indicator of tissue blood supply.^34^ Sahani et al.'s investigation^35^ revealed a significant positive correlation between tumor blood volume and MVD, suggesting that AEF measurements can indirectly reflect the level of MVD in lesions. In our study, AEF emerged as an independent predictor for distinguishing PA and atypical PCA, exhibiting commendable diagnostic value in both the training and test cohorts. The AUCs in the training and test cohorts were 0.703 and 0.708, respectively. The study results indicated that atypical PCA exhibited a higher AEF than PA. Several prior studies have reported that tumor metastatic lymph nodes (LNs) typically display higher AEF compared to non‐metastatic LNs.^34^, ^36^, ^37^ This heightened AEF in metastatic LNs is attributed to increased angiogenesis and a rich blood supply, resulting in early and significant arterial enhancement. Given the relatively dense arrangement of cells and rich capillary network in PCA, it often exhibits a higher degree of early enhancement in the arterial phase compared to PA, consistent with the findings related to AEF in our study.^27^ However, the diagnostic efficacy of AEF alone in both the training and test cohorts in this study was relatively modest. The author speculates that this may be attributed to the presence of epithelial components in some PA,^38^ causing significant arterial enhancement at an early stage and minimizing the difference in AEF between PA and atypical PCA. Additionally, akin to the results in the aforementioned studies, the difference in Hematocrit (HCT) between the training and test cohorts in this study did not achieve statistical significance.

Within the realm of clinical and CT features, age and margin emerged as independent predictors for distinguishing between PA and atypical PCA. This aligns with the findings of Yu et al.,^39^ who reported similar results in their investigation into the differentiation of benign and malignant PTs. Several studies have utilized CT imaging to discern benign PTs, showcasing high diagnostic performance.^14^, ^39^, ^40^ Although the diagnostic efficiency of the combined model in this study is somewhat lower than certain models in the aforementioned omics‐related studies, the current application of radiomics in clinical practice remains intricate, characterized by substantial labor and economic costs. Furthermore, given the rarity of PTs and the lack of extensive data support, diagnostic models constructed based on radiomics still grapple with issues of stability and robustness. Consequently, their results remain contentious and challenging to implement in clinical settings. In contrast, the measurement of ECV and AEF on CT images presents a more straightforward, expedient approach with good repeatability. Modern CT machines are equipped with automated functions for calculating ECV and AEF, rendering them more widely utilized and convenient in clinical practice.

This study carries certain potential limitations. Firstly, the inherent constraints of a retrospective study design may impact the robustness of the findings. Secondly, this research represents the inaugural endeavor to employ ECV and AEF for the identification of PTs, necessitating additional investigations and data to validate the experimental outcomes. Furthermore, the relatively limited number of cases involving atypical PCA in this study poses an additional limitation. Thirdly, in this study, we collected ROI through manual delineation, which is time‐inefficient. However, some researchers have successfully implemented automatic segmentation of the parotid gland using artificial intelligence software, achieving excellent accuracy compared to manual segmentation.^41^, ^42^ Therefore, in future studies, we will also try to perform automatic segmentation based on a deep learning architecture. Lastly, the extended time span covered by the cases and the utilization of diverse types of CT scanners introduce the possibility of influencing model construction and performance, thus requiring acknowledgment.

In conclusion, this study introduces an innovative approach by utilizing ECV and AEF for the differential diagnosis of PA and atypical PCA, yielding promising results. The integration of clinical and imaging features into a comprehensive combined model proves successful, significantly enhancing diagnostic performance. This novel and efficient tool contributes to accurate and non‐invasive preoperative diagnoses for patients.

## AUTHOR CONTRIBUTIONS

**Zhen‐Yu Xu:** Conceptualization (lead); data curation (lead); formal analysis (lead); methodology (lead); visualization (lead); writing – original draft (lead). **Lin‐Wen Huang:** Conceptualization (equal); data curation (equal); methodology (supporting). **Yun‐Jun Yang:** Data curation (supporting); visualization (supporting); writing – review and editing (supporting). **Zhi‐Ping Cai:** Data curation (equal). **Mei‐Lin Chen:** Data curation (equal). **Rui‐Liang Lu:** Investigation (supporting); supervision (supporting); validation (supporting). **Yong‐Xi Ouyang:** Data curation (supporting). **Zhen‐Kai Hong:** Data curation (supporting). **Wei‐Jun Huang:** Project administration (supporting); supervision (supporting). **Zhi‐Feng Xu:** Conceptualization (supporting); formal analysis (supporting); investigation (supporting); methodology (supporting); project administration (lead); supervision (lead); validation (lead); visualization (supporting); writing – review and editing (lead).

## FUNDING INFORMATION

This study has received funding by The Guangdong Basic and Applied Basic Research Foundation under Grant (2019A1515110976), Foshan Science and Technology Bureau Project (2220001003972) and Foshan 14th Five‐Year Plan Key Discipline Foundation (Grant No. FSGSP145036).

## CONFLICT OF INTEREST STATEMENT

The authors of this manuscript declare no relationships with any companies, whose products or services may be related to the subject matter of the article.

## Data Availability

The data that support the findings of this study are available on request from the corresponding author. The data are not publicly available due to privacy or ethical restrictions.
